# Dating the time of viral subtype divergence

**DOI:** 10.1186/1471-2148-8-172

**Published:** 2008-06-09

**Authors:** John D O'Brien, Zhen-Su She, Marc A Suchard

**Affiliations:** 1 Department of Biomathematics, UCLA, Los Angeles, CA, 90095, USA; 2 Department of Mathematics, UCLA, Los Angeles, CA, 90095, USA; 3 State Key Lab for Turbulence and Complex Systems College of Engineering, Peking University Beijing 100871, PR China; 4 Department of Human Genetics, UCLA, Los Angeles, CA, 90095, USA; 5 Department of Biostatistics, UCLA, Los Angeles, CA, 90095, USA

## Abstract

Precise dating of viral subtype divergence enables researchers to correlate divergence with geographic and demographic occurrences. When historical data are absent (that is, the overwhelming majority), viral sequence sampling on a time scale commensurate with the rate of substitution permits the inference of the times of subtype divergence. Currently, researchers use two strategies to approach this task, both requiring strong conditions on the molecular clock assumption of substitution rate. As the underlying structure of the substitution rate process at the time of subtype divergence is not understood and likely highly variable, we present a simple method that estimates rates of substitution, and from there, times of divergence, without use of an assumed molecular clock. We accomplish this by blending estimates of the substitution rate for triplets of dated sequences where each sequence draws from a distinct viral subtype, providing a zeroth-order approximation for the rate between subtypes. As an example, we calculate the time of divergence for three genes among influenza subtypes A-H3N2 and B using subtype C as an outgroup. We show a time of divergence approximately 100 years ago, substantially more recent than previous estimates which range from 250 to 3800 years ago.

## Background

Precise estimates are sorely lacking for dating the emergence and divergence of viral subtypes. Improved estimates equip epidemiologists and virologists to begin to correlate these important establishing events with historical demographic changes, geographical invasions and zoonoses, the transferring of a virus from one host species to another [7,1,25]. For example, archeological sequence data can furnish accurate dates and show that substantial genomic changes associate with geographical invasion and zoonosis [14,17]. Further, the recent availability of viral gene sequences sampled at a pace commensurate with their rate of nucleotide substitution vastly augments the ability to rigorously infer the time scale of phylogenies and hence determine the time of the most recent common ancestor (TMRCA) for different viral types [18,26,6].

Systematic studies characterize the substitution process and substitution rate process of several classes of viral subtypes in, for example, Dengue, influenza subtype A, human immunodeficiency virus (HIV) and the virus responsible for sudden acute respiratory syndrome (SARS). For the last three viruses, a unique zoonotic transfer appears to co-occur with substantial changes in both the composition of nucleotides and amino acids as well as alterations in the rate of nucleotide substitution [15,14,1]. In Dengue, where a single subtype simultaneously inhabits two hosts (humans and *Aedes aegypti*) in a persistent zoonotic process, the introduction of the virus to new geographical environments associates with a dramatic increase in sequence diversity [25]. Unfortunately, no studies thus far analyze the rate of nucleotide substitution during either geographical invasion or zoonosis. Consequently, studies of the date of origins of viral subtypes must use strong *a priori *assumptions on the rate structure of nucleotide substitution.

Two primary methods find use to date the time of viral subtype divergence. The most commonly employed approach determines the divergence time of subtypes using a molecular clock assumption (MCA) over an entire phylogeny [18,21,5,26]. In its strict formulation, the MCA posits a proportional relation between the number of substitutions and the intervening time period over the entire phylogeny. Looser forms of MCAs require only that the proportionality hold along individual branches, with the rates across branches drawn from a pre-specified distribution [5]. Committed to some variant of the MCA, current algorithms then estimate the rate of nucleotide substitution over all taxa in a given set. Consequently, these methods provide inference most suitable for situations where sequence evolution follows a MCA (e.g. influenza A-H3N2 in human hosts, as in [9]) or deviates from the MCA homogenously in time (e.g. perhaps influenza A in wild fowl, see [3]). In considering divergence events between viral subtypes, even when the MCA well-approximates nucleotide substitution within a given subtype, the above methods may incorrectly infer the time of divergence across subtypes. By either assuming that a single rate of nucleotide substitution holds for the region preceding the common ancestor of each subtype or by smoothing the rate of nucleotide substitution over clades with different numbers of taxa, the adherence to a MCA prevents direct inference of the rate during subtype divergence.

Suzuki and Nei (2002) propose an alternative, more heuristic method of estimation to counteract the problem of differing rates of substitution before and after zoonotic events [23,25]. In these studies, the evolutionary models draw a distinction between the rate of substitution within a given subtype and the rate of substitution between subtypes. However, trouble arises since there are no methods for estimating the latter quantity. Consequently, the models assume that the rate of substitution for portions of the phylogeny between the subtypes equals the mean rate in the initial host species population. For instance, in dating the time of divergence between influenza B hemagglutinin and influenza C hemagglutinin-esterase, Suzuki and Nei use the rate of amino acid substitution for water fowl for the portions of the phylogeny previous to the TMRCA of these two proteins [23]. While this method may accurately reflect the rate within avian and human hosts, it neglects whatever additional changes in the rate of substitution are due to the process of zoonotic adaptation, likely leading to a substantial underestimation of the date of the TMRCA.

The study here focuses on influenza, although the techniques are readily applied to other rapidly evolving organisms. Influenza has three types, A, B and C, classified based on serological analysis. To date, only type A sequences have been demonstrably associated with global pandemics [4]. Since modern surveillance began in the 1930s, type B has only been responsible for mild epidemics while type C has been nearly asymptomatic in human infection. Several subtypes of A, notably H1N1 and H3N2, are currently co-circulating in the human population. As the H1N1 and H3N2 subtypes may be as divergent from each other as they are from types B and C, we will refer to all types and subtypes simply as subtypes for the remainder of this paper. We select for this study three genes, coding for hemagglutinin (HA), the matrix protein (MP) and the non-structural protein (NS) responsible for interfering with host immune response. Subtype C has a hemagglutinin-esterase gene that is analogous to the hemagglutin gene in other subtypes [1]. We hence refer to the hemagglutinin gene generally and the hemagglutinin-esterase gene when referring specifically to the subtype C sequences.

We present a simple estimation tool to determine the date of divergence among viral subtypes that overcomes the difficulties encountered with use of the MCA by measuring the pairwise rate of substitution between taxa. Our estimator derives from the triplet statistic developed in [26,22,13], where each sequence member of the triplet draws from a different subtype. In this manner, we generate from each triplet an estimate of the rate of nucleotide substitution between the most recently diverged subtypes, and consequently provide an estimate of the TMRCA. This circumvents the problems posed by earlier methods by directly estimating the pairwise rate of nucleotide substitution over the set of pairs of sequences straddling the subtype divergence without any further rate assumptions other than the existence of a mean. However, this method is only capable of determining the rate between two subtypes where a third, more distantly related, subtype functions as an outgroup. This method thus trades the *ad hoc *rate assumptions of the previous methods with two implicit conditions: (i) that subtypes have a unique divergence and (ii) a third, comparable subtype is available to serve as an outgroup. In exchange, we arrive at a precise statistical measure of the TMRCA that converges as the number of taxa increases and is robust to the balancing of the numbers of taxa between different subtypes. We show that applying this method to dating the divergence of influenza subtypes A-H3N2 and B gives a time of divergence approximately 100 years before present, substantially more recent than previous estimates.

## Methods

**Figure 1:**
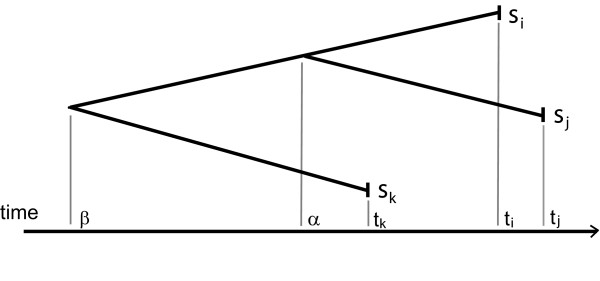
**The phylogenetic relationships between three sequences *s*_*i*_, *s*_*j *_and *s*_*k*_, sampled on dates *t*_*i*_, *t*_*j *_and *t*_*k *_respectively.** The time of most recent common origin of *s*_*i *_and *s*_*j *_is *α*. The time of the most recent origin of all sequences is *β*.

To calculate the rate of nucleotide substitution, we require a measurement of the number of nucleotide substitutions occurring in a given time interval. Starting from a given set of aligned sequences {*s*_1_, ..., *s*_*n*_} for *n *taxa, we define the pairwise distance in number of substitutions to be the estimates {*K*_*ij*_} under a given model of nucleotide substitution. Naturally the unobservable true values {*D*_*ij*_} of the pairwise distances differ from their estimates {*K*_*ij*_}. To understand this difference, we associate each *D*_*ij *_with an error *ε*_*ij *_and assume that *ε*_*ij *_tends to zero as sequence lengths increase without bound. We further assume that the covariance between errors, cov(*ε*_*ij*_; *ε*_*mn*_), is bounded and known. For time measurements, we assume that each sequence is labeled by a sampling time *t*_*i *_given in consistent units. Since we know only the sampling time of a given sample up to the unit of time reported (day, month, year) we posit an uniform error *ν*_*i *_~ *U *[0, 1] underlying each *t*_*i *_over the unit sampling interval. To complete the error structure specification we force the two forms of error (*ν*_*i *_and *ε*_*ij*_) to be independent. Finally, for a set of three sequences (*s*_*i*_, *s*_*j*_, *s*_*k*_) and their associated pairwise distances, we enforce a fixed topology among sequences, as shown in Figure 1, via methods outlined in [26]. We augment the topology with the observed sampling times of the three sequences, *α*, the divergence time between the two sequences of interest and *β*, the divergence time of all sequences. When necessary for clarity, we write *α*_*ij *_to indicate the true time of divergence between sequences *i *and *j*.

Under our triplet method, we aim to estimate the true rate of nucleotide substitution, *p*_*ij*_, between sequences *s*_*i *_and *s*_*j *_with an unobserved error *δ*_*ij*_. With respect to outgroup sequence *k*, an unbiased estimate ${\hat{p}}_{ij}^{\left(k\right)}$ is

(1) $${\hat{p}}_{ij}^{\left(k\right)}=\frac{K_{ik}-K_{jk}}{t_{i}-t_{j}}\times (1-\Delta_{ij}),$$

where the factor $\Delta_{ij}=1+(t_{i}-t_{j}-1)\cdot (t_{i}-t_{j})\cdot \log(\frac{t_{i}-t_{j}}{t_{i}-t_{j}-1})+(t_{i}-t_{j}+1)\cdot (t_{i}-t_{j})\cdot \log(\frac{t_{i}-t_{j}}{t_{i}-t_{j}+1})$ corrects for bias resulting from the time sampling error structure (see Appendix for derivation). We superscript ${\hat{p}}_{ij}^{\left(k\right)}$ to denote its weak dependence on outgroup sequence *k*. Dependence is weak as the path of evolution from *t*_*k *_to *α *is shared between the paths from sequence *k *to both sequence *i *and sequence *j *and hence largely cancels out in Equation 1. We make this transparent in the following derivation. For brevity, we consider only unobservable true values, ignoring error terms. Let *u *be the location on the triplet in Figure 1 corresponding to time *α *and let *p*_*xy *_be the true rate along the path connecting locations *x *and *y*. Then, as distance is rate multiplied by time, we have

(2) $$\begin{array}{c} D_{ik}=p_{ij}(\alpha -t_{i})+p_{ku}(\beta -t_{k}+\beta -\alpha )\\ D_{jk}=p_{ij}(\alpha -t_{j})+p_{ku}(\beta -t_{k}+\beta -\alpha ). \end{array}$$

Subtracting the first equation from the second equation yields *D*_*ik *_- *D*_*jk *_= *p*_*ij*_(*t*_*j *_- *t*_*i*_), which is equivalent to Equation 1. This derivation makes clear that the estimator (1) measures the rate along the path from sequence *i *to sequence *j*, with only incidental dependence on sequence *k*.

The variance for the estimator (1) is well approximated by

(3) $$\mathrm{var}({\hat{p}}_{ij}^{\left(k\right)})\approx \mathrm{var}(\varepsilon_{ij})\cdot {\left(1-\Delta_{ij}\right)}^{2}.$$

Further, we can estimate the time of subtype divergence *α *(Figure 1) between sequences via

(4) $${\hat{\alpha }}_{ij}^{\left(k\right)}=\frac{1}{2}\left(t_{i}+t_{j}-1-\frac{K_{ij}}{{\hat{p}}_{ij}^{\left(k\right)}}\right).$$

We note that the term *t*_*i *_+ *t*_*j *_- 1 is used rather than *t*_*i *_+ *t*_*j *_to account for the expected error coming from the uniformly distributed *ν*_*i *_and *ν*_*j*_.

As nucleotide data increases without bound, *K*_*ij*_* → D*_*ij *_and ${\hat{p}}_{ij}^{\left(k\right)}$ → *p*_*ij*_, ensuring that ${\hat{\alpha }}_{ij}^{\left(k\right)}$ → *α*_*ij*_. For finite sequence lengths, this relation ensures that $\alpha_{ij}-{\hat{\alpha }}_{ij}\sim \frac{\varepsilon_{ij}}{p_{ij}}$. To gain an understanding of this estimator, we note that with a standard model of substitution (e.g. JC69, HKY85), a rate of substitution of 10^-4 ^(s/s/yr) and a sequence of 2000 nucleotides, the above estimator yields a standard error of approximately 23 years [20].

The above derivations express our rate and time estimates for a single triplet of sequences. We now consider estimates that combine information across multiple representative sequences from each subtype. For discussion, we label subtypes *A*, *B *and *C *(which are only incidentally the same as the labels for influenza) and we assume the topology in Figure 1 for these groups. We let *n*_*r*_, where *r *∈ {*A*, *B*, *C*}, count the number of sequences in each group. Then when choosing triplets (*s*_*i*_, *s*_*j*_, *s*_*k*_), there exist *n*_*A *_· *n*_*B *_· *n*_*C *_choices, from which we form a single rate estimate ${\hat{p}}_{AB}^{\left(C\right)}$ that appropriately averages the set {${\hat{p}}_{ij}^{\left(k\right)}$: *i *∈ *A*, *j *∈ *B*, *k *∈ *C*}: This works as all triplets have been selected to contain the divergence point between *A *and *B*. In order to make our estimate robust to outliers and noise, we employ an inverse variance weighting [12]. This standard weighting de-emphasizes the contribution from estimates with high variance, providing significant protection against estimates with little information. Using this weighting, the estimate becomes

(5) $${\hat{p}}_{AB}^{\left(C\right)}=\frac{1}{P}\sum_{i,j,k}\frac{{\hat{p}}_{ij}^{\left(k\right)}}{\mathrm{var}[{\hat{p}}_{ij}^{\left(k\right)}]},$$

where *P *is the sum of the inverse variance of each estimate, ${\hat{p}}_{ij}^{\left(k\right)}$.

The global divergence time estimator $\hat{\alpha }$ is a variance-weighted average over {${\hat{\alpha }}_{ij}^{\left(k\right)}$} sub-stituting ${\hat{p}}_{AB}^{\left(C\right)}$ for the rate,

(6) $$\hat{\alpha }=\frac{1}{P_{\alpha }}\sum_{i,j,k}\frac{{\hat{\alpha }}_{ij}^{\left(k\right)}}{\mathrm{var}[{\hat{\alpha }}_{ij}^{\left(k\right)}]}$$

where *P*_*α *_is the sum of the inverse variance of each estimate, ${\hat{\alpha }}_{ij}^{\left(k\right)}$. Having found $\hat{\alpha }$, we estimate its variance by a bootstrap resampling of sequences from each subtype [8].

The computational efficiency of this estimator is on the order *O*(*n*^3^) for a tree of *n *taxa. This is natural as each of the initial rate estimates ${\hat{p}}_{ij}^{\left(k\right)}$ is composed of information concerning three taxa. While the growth of computational expense in the number of taxa may appear unpleasant, in practice this algorithm is both fast and stable, owing to the absence of costly optimization procedures for parameter inference, and is able to handle data sets of thousands of taxa. The authors detail the computational efficiency of a similar statistic in [26]. As an example, for the data presented below all computations required only a few seconds on a desktop computer.

## Data and Results

**Figure 2:**
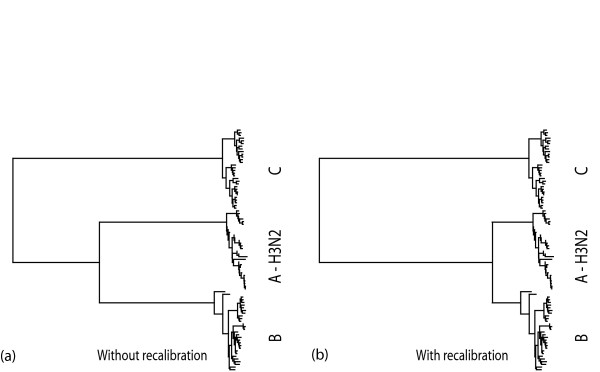
**Phylogeny of 60 influenza hemagglutinin nucleotide sequences from subtypes A-H3N2, B, and C.** We reconstruct the phylogeny in (a) under a strict molecular clock via TREBLE [26]. The phylogeny in (b) is the same tree as in (a) with the divergence time between subtypes A and B recalibrated relaxing the molecular clock. (a) Without recalibration (b) With recalibration.

We demonstrate the advantage of our triplet estimator through analysis of influenza A-H3N2/B subtype divergence using the hemagglutinin (HA), matrix protein (MP) and non-structural (NS) genes. Each analysis is performed on 60 gene sequences constructed from 20 genomes each drawn from influenza subtypes A-H3N2, B and C. We download these data along with their dates of sampling from the Los Alamos Influenza Database [16]. We perform sequence alignment using ClustalX [24, version 1.8]. For consistency with previous studies of A-H3N2 HA evolution, we use the HKY model of nucleotide substitution [10]. We use the TREBLE algorithm, which implements a MCA, on sets of sequences solely drawn from a single subtype to derive within-subtype rates. The phylogenetic tree, generated by TREBLE, for the HA gene is depicted in Figure 2(a). We infer similar trees for the MP and NS genes. We calculate variances for both MCA and pairwise rate estimates using 200 bootstrap iterates. All dates are listed as years in the common era.

**Figure 3:**
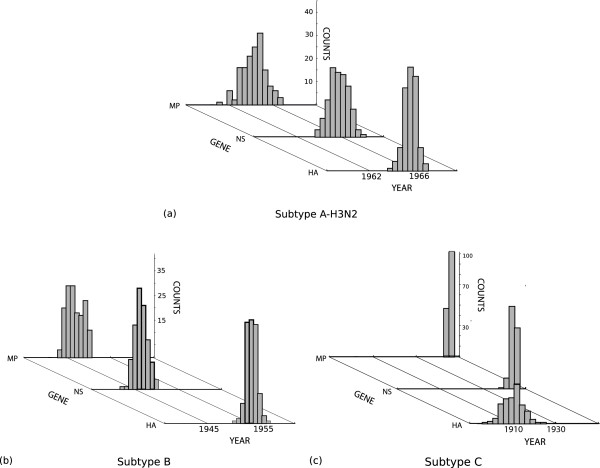
**Histograms of the time of most recent common ancestor for subtypes A-H3N2, B and C, respectively, derived from molecular clock estimates on hemagglutinin (HA), matrix (MP) and nonstructural (NS) gene sequences. **(a) Subtype A-H3N2 (b) Subtype B (c) Subtype C.

Consistent with previous studies [1-3], rates vary substantially both among genes and among subtypes. We record rates as a point estimate (± standard error). For the HA gene, subtype A-H3N2 shows a rate of nucleotide substitution of 3.21 (± 0.43) × 10^-3^s/s/yr. This rate is slightly lower than those recorded in previous studies although within the margin of error [26]. For subtype B, the rate of nucleotide substitution is 2.31 (± 0.37) × 10^-3^s/s/yr, which is higher than previous estimates although also within the margin of error [23], and for subtype C, the rate is 0.68 (± 0.18) × 10^-3^s/s/yr. For the MP gene, rates are generally lower than those for HA. The subtype A-H3N2 rate is 1.57 (± 0.38) × 10^-4^s/s/yr. The subtype B rate is 2.20 (± 0.48) × 10^-3^s/s/yr and the subtype C rate is 1.31 (± 0.33) × 10^-3^s/s/yr. Lastly, for the NS gene, the rates are similar to those of the MP gene. The subtype A-H3N2 rate is 2.14 (± 0.25) × 10^-3^s/s/yr, the subtype B rate is 1.92 (± 0.20) × 10^-3^s/s/yr, and the subtype C rate is 1.68 (± 0.51) × 10^-3^s/s/yr. Table 1 presents these results. Figure 3 provides histograms of the bootstrap distributions for all three genes and subtypes.

**Table 1:** Within-subtypes rates of nucleotide substitution for hemagglutinin (HA), matrix (MP) and non-structural (NS) genes for subtypes A-H3N2, B and C.

Subtype		A-H3N2	B	C
Gene:	HA	3.21 ± 0.43	2.31 ± 0.37	0.68 ± 0.18
	MP	1.57 ± 0.38	2.20 ± 0.48	1.31 ± 0.33
	NS	2.14 ± 0.25	1.92 ± 0.20	1.68 ± 0.51

Rates are given in × 10^-3^substitutions per site per year ± one standard error.

Assuming a molecular clock within a subtype and with the rates above, we generated the corresponding dates of the TMCRA. Figure 3 shows histograms of the TMRCA estimates for different genes and subtypes. All genes are similar in dating the TMRCA for A-H3N2 to approximately 1965 (1964, 1965, and 1962 for HA, MP and NS genes, respectively). These dates are consistent with the emergence of the A-H3N2 subtype into global circulation during the 1968 pandemic [1]. Both the MP and NS genes date the TMRCA of subtype B to 1943, while the HA rate places the TMRCA at 1953. This latter value is inconsistent with the influenza B sub-epidemics of 1950–51 but is consistent with the emergence of the more lethal Victoria strain of influenza B in 1953 [11]. Each of these estimates has a standard error of approximately 2 years and so these discrepancies may be accounted by measurement uncertainty. The 10 year gap between the TMCRA suggested by the different genes can be explained by a reassortment event. Finally, the TMRCA of subtype C is calculated as 1952 and 1953 by the MP and NS genes, respectively, while the HA gene places the TMCRA at 1906. This nearly half century discrepancy suggests that the subtype C HA gene experienced a markedly different evolutionary history than either the MP or the NS gene. A biologically plausible explanation would be a reassortment event. Another possible explanation is that non-MCA rate behavior has lead to substantial bias in dating the TMRCA.

**Figure 4:**
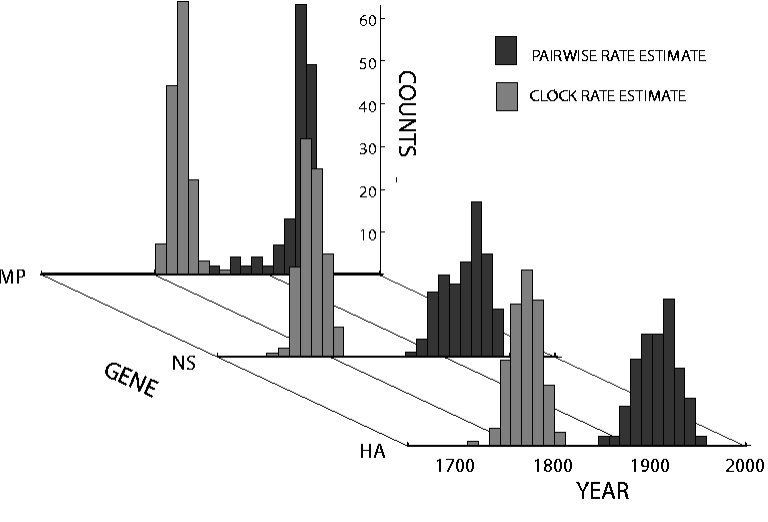
Histograms of the time of most recent common ancestor of subtypes A-H3N2 and B, derived from molecular clock estimates (light grey) and pairwise estimates (dark grey) on hemagglutinin (HA), matrix (MP) and nonstructural (NS) gene sequences.

We now compare the results from pairwise rate estimates across subtypes A-H3N2 and B with those from application of the MCA to the same data. These results are summarized in Table 2 and Figure 4. Using the triplet method developed above, data from the hemagglutinin gene yields a pairwise rate of substitution between subtypes A-H3N2 and B, ${\hat{p}}_{A-H3N2,B}^{C}$, of 8.66 (± 0.26) × 10^-3 ^s/s/yr. Via Equation 3, and averaging over all possible pairs of sequences (*s*_*i*_, *s*_*j*_) ∈ {A-H3N2, B}, the date of divergence between the two subtypes is then 1905 (± 20) years. Under a molecular clock, the substitution rate for HA over both subtypes A-H3N2 and B is 3.10 (± 0.37) × 10^-3 ^s/s/yr, implying a TMCRA at 1789 (± 12.5). A similar pattern emerges for the MP gene. The pairwise rate of substitution is 6.46 (± 1.31) × 10^-3 ^s/s/yr, yielding a TMRCA at 1912 (± 18) years. The MCA rate of substitution is 2.13 (± 0.35) × 10^-3 ^s/s/yr with a corresponding TMRCA of 1759 (± 15). Finally, for the NS gene, the pairwise rate of substitution is 7.95 (± 0.25) × 10^-3 ^s/s/yr, leading to the TMRCA as 1902 (± 19) years. Under the MCA, the rate of substitution is 2.22 (± 0.38) × 10^-3 ^s/s/yr with a corresponding TMCRA of 1777 (± 14). Summarizing these results, we find that the pairwise rate estimates are consistent in placing the TMRCA at approximately 1905 while the MCA rate estimates correspond to a TMRCA at approximately 1775. This discrepancy between the two sets of estimates of the TMRCA likely owes to the inability of the MCA to integrate information from the period of evolution between the two subtypes, leading to a substantial underestimate of the rate of substitution, and consequent underestimation of the date of the TMRCA.

**Table 2:** Across-subtype rates of nucleotide substitution between subtypes A-H3N2 and B for hemagglutinin (HA), matrix (MP) and non-structural (NS) genes.

Method:		Pairwise	MCA
Gene:	HA	8.66 (0.26)	3.10 (0.37)
	MP	6.46 (1.31)	2.13 (0.35)
	NS	7.95 (0.25)	2.22 (0.38)

Rates are given in × 10^-3 ^substitutions per site per year ± one standard error.

## Discussion

We present a new method for ascertaining the rate of nucleotide substitution between subtypes and apply this method together with traditional MCA methods to date the divergence of influenza subtypes A-H3N2, B, and C. We use three genes, HA, MP and NS, to date two types of divergence events: the time of the most recent common of each subtype and the time of divergence between two subtypes, A-H3N2 and B. For the former event type, we show that the three genes are loosely consistent in their dating of the TMRCA of the subtypes, with the notable exception of the HA-derived estimate of subtype C's TMRCA approximately 50 years before the MP- and NS-derived estimates. This discrepancy may indicate either that subtype C's hemagglutinin-esterase gene engaged in a biologically significant event, such as reassortment, or that MCA estimation does not adequately model the evolution of the gene.

For the divergence between subtypes A-H3N2 and B, previous studies using the MCA generally place a time of divergence of several hundred years ago, ranging from the 16th to early 19th centuries. Other analysis have yielded estimates of 3600 years ago [23]. In the current study, application of the MCA yielded estimates in the last half of the 18th century. However, applying the pairwise rate estimate developed above we find uniformly, across genes, that the divergence likely occurred in the very early 20th century. The discrepancy between these two measures is likely due to the increased modeling flexibility of the pairwise rate estimate relative to the MCA.

This discrepancy between the rates and corresponding TMCRA estimates has important biological consequence. The phylogenetic divergence between subtype A-H3N2 and B corresponds to a subspeciation event for the virus. The results in this study indicate that the process of speciation is not neutral but instead a period of rapid and intense genetic change. The three genes studied here consistently show large acceleration in the rate of nucleotide substitution for the divergence period relative to the rates observed within a stable subtype. This study gives strong evidence that, at least for influenza viral subtype divergence, the process of subspeciation is associated not just with large genomic changes but also with an accelerated, finite process of adaption.

Assuming that the more recent estimate is correct, a subsequent question is whether or not a pandemic or epidemic associates with subtype A-H3N2/B divergence. In the twentieth century, all influenza pandemics associate with the emergence or reemergence of subtypes (A-H1N1 in 1918, A-H2N2 in 1957 and A-H3N2 in 1968). Serological analysis indicates that the 1897 pandemic was likely due to subtype A-H2N2. However, the pandemic of 1900 is of uncertain type, although it is commonly reported in the literature as being due to A-H3N2 [4]. The above analysis suggests that it is possible to postulate that the cause of this pandemic is due to the emergence of subtype A-H3N2 or B.

As noted above, we condition the results presented here on a specific sequence alignment. As the question under consideration concerns the divergence of specific genes and proteins over a (presumably) long time scale, the capacity to generate reasonable alignments diminishes with increasing time of divergence between types, conditional on the rate of substitution. We find that for the hemagglutinin gene, a proportion of sequence alignments support the split of subtype B from subtype C after the split between subtypes A-H3N2 and B, in opposition to the topology enforced in our analysis. Hence, to some unknown degree, our analysis is necessarily biased by the choice of alignment. This suggests that improved dating can be found by integrating estimation procedures over an ensemble of alignments [19].

The pairwise estimate method presented above is accurate in the scale

(7) $$\bar{T}\cdot n\cdot p\sim O(1),$$

where $\bar{T}$ is the total time over the phylogeny and *p *is mean rate over the phylogeny [26]. This relation dictates that as divergence events become more remote the ability of the triplet method to resolve the time of divergence diminishes. While this limit prohibits the calculation of remote divergence events, the example presented above lies within the appropriate scale.

In place of a specific MCA, the estimates presented here directly calculate the rate of substitutions between taxa from different viral subtypes. As such estimates span paths between subtypes, they simultaneously capture the rate evolution along branches both within and between subtypes. From these estimates, we are able to directly infer the time of divergence between subtypes. As a trade-off for limited MCAs, the method requires an outgroup subtype to function as an origin relative to the subtypes under consideration. We feel that the triplet method provides a simple and widely applicable way to calculate the dates of divergence of rapidly evolving organisms without the pitfalls of the MCA.

## Conclusion

We present a simple method for calculating the time of viral subtype divergence that does not assume a molecular clock over the entire phylogeny. Additionally, the estimator of this method, a weighted sum of pairwise estimates, furnishes a defined variance for the time of the most common ancestor between subtypes. As a tradeoff for this increased precision, the structure of the triplet statistic requires an outgroup set of sequences, usually a closely related subtype. We apply this estimator to the case of influenza subtype divergence, considering three genes. We show that the estimated divergence time of subtypes A-H3N2 and B is more than a century later than those calculated with a molecular clock.

## Authors' contributions

JDO'B collected the data, designed and performed the study and wrote the initial manuscript. ZSS provided extensive review of the study design and provided assistance in revising the manuscript. MAS contributed extensive work in reviewing and revising the manuscript.

## Appendix

Initially, one might define an estimator ${\tilde{p}}_{ij}^{\left(k\right)}$ of the conditional pairwise rate $p_{ij}^{\left(k\right)}$ to be

$${\tilde{p}}_{ij}^{\left(k\right)}=\frac{K_{ik}-K_{jk}}{t_{i}-t_{j}},$$

that has been previously used in the paper outlining the TREBLE algorithm [26], and originates in [13]. However, this apparently natural statistic is substantially biased when the sampling times of sequences *i *and *j *are close. To be seen in the following derivation, this bias is the result of the time sampling error structure.

As the true value of the rate of substitution is given by

$$p_{ij}^{\left(k\right)}=\frac{D_{ik}-D_{jk}}{t_{i}-\nu_{i}-t_{j}+\nu_{j}}=\frac{K_{ik}-\varepsilon_{ik}-K_{jk}+\varepsilon_{jk}}{t_{i}-\nu_{i}-t_{j}+\nu_{j}},$$

we then have an expression for the error:

$$\begin{array}{lll} {\tilde{p}}_{ij}^{\left(k\right)}-p_{ij}^{\left(k\right)} & = & \frac{K_{ik}-K_{jk}}{t_{i}-t_{j}}-\frac{K_{ik}-\varepsilon_{ik}-K_{jk}+\varepsilon_{jk}}{t_{i}+\nu_{i}-t_{j}-\nu_{j}}\\ & = & \frac{\left(\nu_{i}-\nu_{j})(K_{ik}-K_{jk})-(t_{i}-T_{j})(\varepsilon_{jk}-\varepsilon_{ik}\right)}{\left(t_{i}-t_{j})(t_{i}-\nu_{i}-t_{j}+\nu_{j}\right)}. \end{array}$$

Taking the expectation yields the bias:

$$E({\tilde{p}}_{ij}^{\left(k\right)}-p_{ij}^{\left(k\right)}=E\left(\frac{\left(\nu_{i}-\nu_{j})(K_{ik}-K_{jk}\right)}{\left(t_{i}-t_{j})(t_{i}-\nu_{i}-t_{j}+\nu_{j}\right)}-\frac{\left(t_{i}-t_{j})(\varepsilon_{jk}-\varepsilon_{ik}\right)}{\left(t_{i}-t_{j})(t_{i}-\nu_{i}-t_{j}+\nu_{j}\right)}\right).$$

Since we assume that the *ν *and *ε *structures are independent, the right side of the equation can be further reduced, yielding

$$E({\tilde{p}}_{ij}^{\left(k\right)}-p_{ij}^{\left(k\right)})={\tilde{p}}_{ij}^{\left(k\right)}\cdot E\left(\frac{\nu_{j}-\nu_{i}}{t_{i}-\nu_{i}-t_{j}+\nu_{j}}\right).$$

Let Δ*t *= *t*_*i *_- *t*_*j*_. The final expectation on the right hand side resolves by direct integration,

$$\begin{array}{lll} E\left(\frac{\nu_{j}-\nu_{i}}{\left(t_{i}-t_{j})(t_{i}-\nu_{i}-t_{j}+\nu_{j}\right)}\right) & = & 1+(\Delta t-1)(\Delta t)\cdot \log(\frac{\Delta t}{\Delta t-1})\\ & & +\;(\Delta t+1)(\Delta t)\cdot \log(\frac{\Delta t}{\Delta t+1})\\ & = & \Delta_{ij}. \end{array}$$

We note that as the sampling time is independent of the rate of nucleotide substitution, the error increases in proportion to the magnitude of the initial statistic. We can then create a new, unbiased statistic by counterbalancing the original statistic with this factor, making a new statistic

$${\hat{p}}_{ij}^{\left(k\right)}={\tilde{p}}_{ij}^{\left(k\right)}\cdot (1-\Delta_{ij}).$$
